# Hydrothermal Corrosion of Double Layer Glass/Ceramic Coatings Obtained from Preceramic Polymers

**DOI:** 10.3390/ma14247777

**Published:** 2021-12-16

**Authors:** Ivana Parchovianská, Milan Parchovianský, Hana Kaňková, Aleksandra Nowicka, Dušan Galusek

**Affiliations:** 1 Centre for Functional and Surface Functionalised Glass, Alexander Dubček University of Trenčín, Študentská 2, 911 50 Trencin, Slovakia; milan.parchoviansky@tnuni.sk (M.P.); hana.kankova@tnuni.sk (H.K.); aleksandra.nowicka@tnuni.sk (A.N.); dusan.galusek@tnuni.sk (D.G.); 2 Joint Glass Centre of the Institute of Inorganic Chemistry Slovak Academy of Sciences, Alexander Dubček University of Trenčín and Faculty of Chemical and Food Technology Slovak University of Technology, Študentská 2, 911 50 Trencin, Slovakia

**Keywords:** polymer derived ceramics, coatings, fillers, stainless steel, hydrothermal corrosion

## Abstract

Polysilazane-based double layer composite coatings consisting of a polymer-derived ceramic (PDC) bond-coat and a PDC top-coat that contains ceramic passive and glass fillers were developed. To investigate the environmental protection ability of the prepared coatings, quasi-dynamic corrosion tests under hydrothermal conditions were conducted at 200 °C for 48–192 h. The tested PDC coatings exhibited significant mass loss of up to 2.25 mg/cm^2^ after 192 h of corrosion tests, which was attributed to the leaching of elements from the PDC coatings to the corrosion medium. Analysis of corrosion solutions by inductively coupled plasma optical emission spectrometry (ICP-OES) confirmed the presence of Ba, Al, Si, Y, Zr, and Cr, the main component of the steel substrate, in the corrosion medium. Scanning electron microscopy (SEM) of the corroded surfaces revealed randomly distributed globular crystallites approximately 3.5 µm in diameter. Energy-dispersive X-ray spectroscopy (EDXS) of the precipitates showed the presence of Ba, Al, Si, and O. The predominant phases detected after corrosion tests by X-ray powder diffraction analysis (XRD) were monoclinic and cubic ZrO_2_, originating from the used passive fillers. In addition, the crystalline phase of BaAl_2_Si_2_O_8_ was also identified, which is in accordance with the results of EDXS analysis of the precipitates formed on the coating surface.

## 1. Introduction

Ferritic stainless steels are frequently used as components in high temperature industrial processes, such as solid oxide fuel cells, exhaust gas elements, waste incineration plants or in the chemical industry, because of their excellent corrosion and oxidation resistance as well as their relatively low cost. However, even stainless steels are susceptible to corrosion under extreme conditions and interact with the environment in which they are used, leading to a deterioration of their physical and mechanical properties which adversely affects their further applicability. Thus, corrosion resistance is one of the most important factors for determining the overall material performance [1]. With the help of protective coatings, the surfaces of metals can be adapted for a specific application and their service life can be drastically extended. Advanced silicon-based ceramics are suitable for operation in aggressive chemical environments, as they resist molten metals, molten salts, hot gases, hot solutions of acids, bases and salts, or supercritical water [2]. For example, their corrosion resistance was shown to be significantly higher than that of metals, even at ultrahigh temperatures [3].

In recent years, the polymer-derived ceramic (PDC) synthesis route has gained attention as an economical and easy approach for producing ceramic coatings by the pyrolysis of suitable organoelemental precursors (preceramic polymers) [4,5,6,7]. The chemical composition of the ceramic after pyrolysis is based on the chemistry of the starting precursors, the preparation conditions (temperature, pressure, pyrolysis atmosphere) and the possible incorporation of other additives or modifiers (fillers, metal alkoxides). Polysiloxanes [8,9], polycarbosilanes [10,11] and polysilazanes [12], characterized by Si–O, Si–N and Si–C chains, allow the preparation of amorphous Si_x_O_y_C_z_, Si_x_C_y_N_z_ and Si_x_C_y_ ceramics after pyrolysis in an inert atmosphere [4,13]. The pyrolysis mechanism involved in the ceramization process is quite complex and involves structural rearrangement and radical reactions that result in the cleavage of Si–H, Si–C and C–H chemical bonds, the release of organic functional groups (CH_4_, C_6_H_6_, CH_3_NH_2_) and the formation of an inorganic network. However, the greatest disadvantage of this approach is high shrinkage of the organosilicon polymer (up to 50 vol. %, depending on the precursor) during pyrolysis [14]. The shrinkage of the precursor can be compensated by adding active (ZrSi_2_ [15] or TiSi_2_ [16]) and/or passive fillers (BN [17], Si_3_N_4_ [18], ZrO_2_ [19], NbC [20] or Al_2_O_3_ [21]). The use of fillers not only reduces the shrinkage of the polymer during pyrolysis, but also allows the preparation of composites with special properties, e.g., high mechanical strength, thermal and electrical conductivity, and improved corrosion and oxidation resistance. When preparing PDCs with the addition of passive fillers, the coefficient of thermal expansion (CTE) of the passive filler must be taken into account, as it affects the total CTE of the final composite. The ability to adapt the CTE is particularly useful in the preparation of coatings on metals, because a large mismatch of the CTE between the coating and a metallic substrate can cause the formation of cracks. This can adversely affect the final mechanical properties and eventually even cause the spallation of the coating from the substrate. The application of polymer precursors represents one of the greatest advantages of PDCs, since all common methods used for application and shaping of conventional polymers can, in principle, also be applied to polymer precursors. Several authors have prepared PDC coatings by spraying [22], dip coating [23,24], the doctor blade method [25], tape casting [26], or spin coating [27]. These features of the PDC can be successfully utilized in the protection of metals, especially steels, used in the construction of heat exchangers in thermal power plants and municipal waste incinerators, which are exposed to the long-term corrosive action of combustion gases at high temperatures. A variety of PDC coatings, such as SiOC [15,16], SiCN [19,28], and SiON [28,29], have been investigated in order to improve the corrosion and oxidation resistance of metallic substrates. However, there is a lack of information about their corrosion behavior in hot, aqueous solutions under subcritical conditions.

The objective of the present study is to demonstrate the possibility of fabrication of environmental barrier coatings from preceramic polymers as well as to asses and discuss their corrosion behavior under hydrothermal conditions at 200 °C. Thick (~40 µm) PDC coatings were prepared, as the increased coating thickness is expected to act as a more robust protective barrier and provide better separation between corrosion medium and the steel substrate. The extent of corrosion in the coatings was monitored by the weight change of the corroded samples, followed by detailed study of the microstructure, phase and chemical composition before and after corrosion tests, and the formation of corrosion products. Analysis of specific elements released from the tested specimens into the corrosion medium was also performed. Our previous work identified this type of PDC glass/ceramic coatings as a suitable coating system for the protection of stainless steel from oxidation in synthetic air and water vapor atmospheres up to 950 °C [30]. The results presented in this paper introduce new information on the corrosion behavior of thick PDC coatings suitable for applications in the field of chemical industry to protect metals against corrosion.

## 2. Materials and Methods

### 2.1. Coatings Processing

The low carbon ferritic stainless steel AISI 441 was selected as a substrate. This material is widely used as a structural material in systems containing water at elevated temperatures and pressures, with the maximum recommended temperature of use 950 °C in dry air. The 1 mm thick steel substrates were dimensioned as 10 × 15 mm^2^ sheets and ultrasonically cleaned in acetone, ethanol, and deionized water (10 min for each) in order to remove the impurities and degrease the surface. Two types of organosilicon precursors were selected: perhydropolysilazane Durazane2250 (Merck KGaA, Darmstadt, Germany) was used to prepare the ceramic interlayer (bond-coat), and Durazane1800 (Merck KGaA, Darmstadt, Germany), liquid organosilazane, was used to prepare the ceramic top-coat. The bond-coat was obtained by the conventional dip-coating technique (dip-coater RDC 15, Relamatic, Glattbrugg, Switzerland): a deposition time of 20 s and a hoisting speed of 0.3 m/min were employed. After deposition, the bond-coat was heat treated in air at 450 °C for 1 h using a heating rate of 5 °C/min (Nabertherm^®^ N41/H, Nabertherm, Lilienthal, Germany). Two prepared preceramic suspensions (denoted as C2c and D4), both containing ZrO_2_ stabilized with 8 mol. % Y_2_O_3_ (8YSZ, Inframat^®^ Advanced Materials TM, Manchester, CT, USA) and a commercial barium aluminosilicate glass (G018-281, Schott AG, Mainz, Germany) as passive fillers, were used to deposit the top-coat onto the pyrolyzed bond-coat by spray-coating (Nordson EFD 781S-SS). The composite glass/ceramic coatings were cured in air at 850 °C at a heating rate of 3 °C/min (Classic 0718E, Prague, Czech Republic) with a 1 h isothermal dwell. In the case of the D4 suspension, a polycrystalline Al2O3-Y2O3-ZrO2 (AYZ) precursor powder prepared by the modified Pechini sol–gel method was used as an additional filler. The filler material was included in the mixture to modify the CTE of the top-coat to closely match the CTE of the steel substrate (11 × 10^−6^/K) and to minimize volume changes during the polymer to ceramic transformation. The compositions of the prepared coatings are listed in Table 1. The detailed preparation of glass/ceramic coatings as well as AYZ precursor powder and its basic characterization was described in our previous work [30,31]. The basic properties of the filler materials are listed in Table 2.

### 2.2. Hydrothermal Corrosion Tests

The hydrothermal corrosion resistance of the prepared glass/ceramic coatings was monitored under quasi-dynamic conditions. The corrosion tests were carried out in stainless steel pressure reactors with an inner Teflon lining that prevented direct contact of the corrosion medium with the steel reactor; 18 ml of deionized water was used as the corrosion medium. Coated samples and uncoated steel substrates were placed in the steel reactors in special holders to ensure maximum contact area of the specimens with the corrosion medium. Quasi-dynamic tests were carried out in a laboratory oven at 200 °C for 192 h. The temperature was selected to fill the gap in knowledge on the corrosion resistance of PDC coatings under subcritical hydrothermal conditions, and to provide new information on the corrosion behavior of the PDC coatings that may be used in e.g., the chemical industry. At defined time intervals (every 48 h), corroded samples and corrosion medium were removed from the reactors. The coated and uncoated samples were rinsed with deionized water, dried in an oven at 100 °C for 2 h and weighed. The pH value of the corrosion medium after each time interval was determined using a pH meter (Mettler ToledoSevenEasy, Columbus, OH, USA). Next, the corrosion medium was stabilized for chemical analysis using nitric acid to pH < 2 at room temperature. Finally, the tested samples were returned to the steel reactors and the corrosion medium was replaced with fresh deionized water. The tests were carried out in three reactors in parallel, i.e., three samples of the same composition were tested under identical conditions in order to ensure the reproducibility of the corrosion results. Moreover, tests without any sample were performed for the given time intervals, providing a blank solution. The concentrations of the elements identified in the blank were subtracted from the concentrations determined in the corrosion medium obtained after the corrosion tests with the coated and uncoated sample.

### 2.3. Characterization Methods

The weight changes of the tested samples as a function of corrosion time were measured using an electronic balance with an accuracy of ±0.0001 g (Axis AD 1000, Gdansk, Poland). The content of metallic elements released from the coated and uncoated samples into the corrosion medium were determined by ICP-OES (Agilent 5100 SVDV, Santa Clara, CA, USA). In this work, the amount of Y, Zr, Si, Ba, Al, and Cr, the main component of the steel substrate, leached from the tested samples was considered for each parallel experiment. The amount of elements released into the solution was calculated according to the Formula (1):

(1) $$Q_{i}^{t}=\frac{\left(c_{i}-c_{blank}\right).V}{S}+Q_{i}^{t-\Delta t}$$

where *Q_i_* is the total amount of leached element *i* in time *t* [mg/m^2^], *c_i_* is the concentration of leached element *i* in time *t* [mg/dm^3^], *c_blank_* is the concentration of element *i* in the blank [mg/dm^3^], *V* is the volume of the corrosion medium [dm^3^], and *S* is the sample surface in contact with the corrosion medium [m^2^]. The microstructure of both uncorroded and hydrothermally corroded samples was examined in detail by SEM (JEOL JSM 7600 F, JEOL, Tokyo, Japan). The chemical composition of the investigated samples and corrosion products formed at the corroded surfaces was determined by EDXS (Oxford instruments, Abingdon, UK). XRD (PANalytical Empyrean DY1098 (Panalytical, BV, Almelo, The Netherlands)) with a Cu anode and an X-ray wavelength of λ = 1.5405 Å over 2θ angles of 10–80° was used to determine the phase composition of the stainless steel and prepared coatings before and after corrosion tests. Diffraction records were evaluated using HighScore Plus (v. 3.0.4, Panalytical, Almelo, The Netherlands) with the use of the Crystallographic Open Database COD2019. Raman spectra of the uncoated steel substrates were recorded in the range of the Raman shift (200–800) cm^−1^ by a RENISHAW inVia Reflex Raman spectrometer (RENISHAW, Wotton-under-Edge, UK).

## 3. Results and Discussion

### 3.1. Weight Changes Measurement

Corrosion may be defined as the physical and chemical alteration of a material due to its interaction with the surrounding environment, which leads to the loss of functional properties of the material of interest. Corrosion may be broadly divided into two modes: active and passive. The active form is characterized by the loss of material in contact with the environment, which is accompanied by the decrease in size and weight of the specimen. The losses can be in the form of gaseous or dissolved species. The passive form involves processes where the material reacts with the environment to form a new condensed phase covering the surface (layer or scale) and is associated with a weight gain of the component [32]. Hence, the weight gain or loss is an important parameter indicating the lifetime prediction and corrosion mechanism involved.

**Figure 1:**
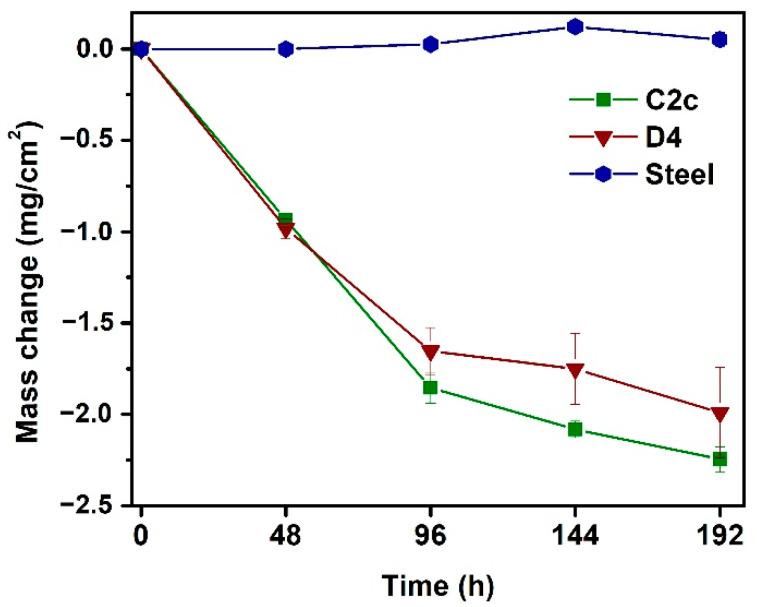
Mass changes measured for coated and uncoated samples tested in deionized water at 200 °C.

Figure 1 displays the time dependencies of the cumulative weight changes normalized with respect to the corroded surface area for the coated and uncoated samples tested in deionized water at 200 °C. Both tested coatings, C2c and D4, exhibited significant mass loss during corrosion tests in deionized water compared to the uncoated steel substrates. This indicates an active corrosion mode, which is a typical behavior of Si-based ceramics upon exposure to a hydrothermal environment [33,34]. The observed weight losses of the coated samples can be explained by the reaction of some of the glass-ceramic matrix components with the deionized water and the subsequent dissolution and release of elements from the coatings. In contrast, the uncoated steel substrates showed a relatively small mass gain after the tests due to the formation of corrosion products on their surface. The largest weight gain of uncoated steel exposed to deionized water was observed after 144 h of corrosion tests, reaching a value of only 0.121 mg/cm^−2^. At the end of the experiment, i.e., after 192 h, the uncoated steel achieved a smaller weight gain of 0.051 mg/cm^2^. In the first 48 h of corrosion tests, comparable values of weight losses were measured for both PDC coatings. After 48 h, the weight loss in the C2c coating was found to be higher than for the D4 coating for the rest of the experiment. The measured weight loss in the D4 coating after 192 h was 1.99 mg/cm^2^. In the C2c coating the weight loss was higher at 2.25 mg/cm^−2^. In the time interval up to 96 h, rapid mass loss in both coatings was observed, followed by a slower rate of mass loss until the end of the corrosion tests. This could indicate that a state of saturation was achieved after 96 h of exposure to deionized water. However, there are two phenomena that influence weight change measurements and the determination of corrosion mechanisms in the present case: the dissolution and release of elements from the coatings that cause a mass loss, and the almost immediate corrosion products formation causing mass gain. Other factors, especially inhomogeneous dissolution in some places (e.g., microcracks in the coatings) with simultaneous precipitation of reaction products and the formation of a passivation layer at other places, could influence the mass loss-time dependencies significantly. Therefore, in this case, we do not consider the mass change measurement to be a definitive parameter for evaluating corrosion mechanisms.

### 3.2. Surface Morphologies of Corroded Samples

**Figure 2:**
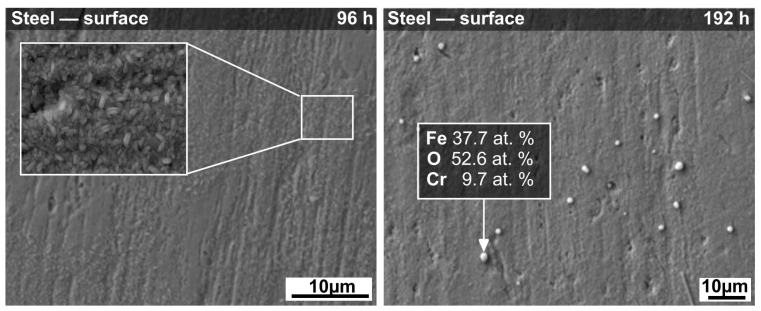
Scanning electron microscopy (SEM) micrographs of uncoated steel surfaces after corrosion tests in deionized water at 200 °C.

SEM analysis was used for a detailed study of the surfaces of uncoated and coated samples after corrosion tests. Visual inspection of the uncoated steel substrates revealed that all tested steel samples exhibited a loss in brightness and the initial shiny silver surface turned into a red-yellowish color after the corrosion tests. Figure 2 shows the surface morphologies of stainless steel substrates without any coating after 96 h and 192 h of corrosion tests. As can be seen in Figure 2 (96 h), most of the surface exhibits thin oxide scales consisting of tiny rod-shaped crystals up to 1 µm in size rather than a continuous layer of corrosion products. After 192 h of corrosion testing, the bare substrate shows a homogeneous corrosion attack and growth of the rod-shaped corrosion products all over the surface. The EDXS analysis (not shown) of these crystals formed at the steel’s surface showed the presence of Fe, Cr, and O, with a small amount of Mn, indicating (Mn, Cr, Fe)_3_O_4_ spinel formation. However, due to the small size of crystals and the thickness of the layer of corrosion products, the EDXS analysis was probably affected by the underlying steel substrate and is, therefore, not considered as a suitable indication of the real composition of the crystallites. SEM examination also revealed a few randomly distributed crystallites with spherical morphology, approximately 2.5 µm in diameter, identified by EDXS analysis as a mixture of iron and chromium oxide (see Figure 2—192 h).

**Figure 3:**
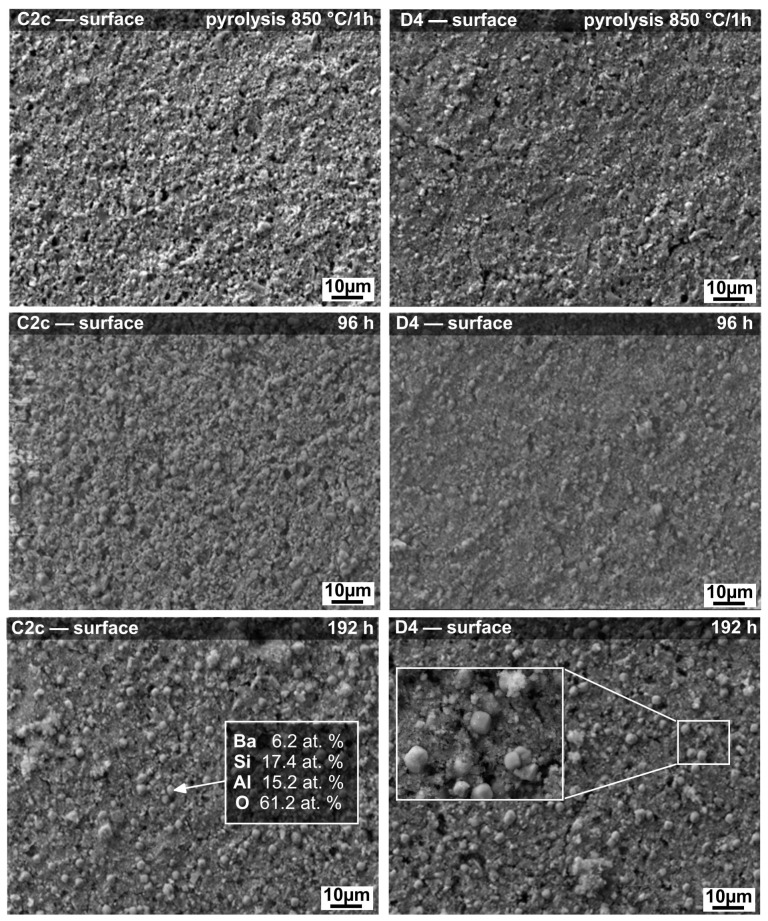
SEM micrographs of C2c and D4 coatings’ surfaces before and after corrosion tests in deionized water at 200 °C.

SEM micrographs of the C2c and D4 coated samples’ surfaces, before and after corrosion tests, are shown in Figure 3. After pyrolysis in air at 850 °C for 1 h, homogeneous and almost dense protective coatings, with only small pores, were prepared. Both coatings, C2c and D4 (Figure 3), showed neither delamination nor significant cracks at the surface. Hence, they were expected to protect the steel substrate against corrosion in deionized water. According to the visual inspection of the coatings after corrosion tests, both compositions showed no signs of corrosion and the coatings adhered very well to the steel substrate. SEM examination of the corroded surfaces (Figure 3) revealed randomly distributed globular crystallites, approximately 3.5 µm in diameter. The precipitates were found to form after 96 h of testing, remaining spherically shaped during the whole experiment. In both coatings, the precipitates were morphologically similar, and from the point of view of their chemical composition, they were identical. The surface of the coating became less smooth with increasing time of exposure to the corrosion medium, which was attributed to crystal growth.

**Figure 4:**
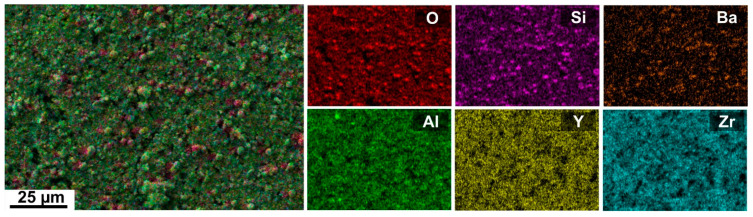
SEM/energy-dispersive X-ray spectroscopy (EDXS) analysis of the surface of the D4 coating after 192 h of corrosion tests in deionized water.

In Figure 4, the morphology of the D4 coating surface, including an EDXS elemental map, is displayed. EDXS analysis of the spherical crystals showed the presence of Ba, Al, Si, and O, indicating the formation of BaAl_2_Si_2_O_8_ precipitates. The EDXS map of the surface of the D4 coating was similar to the C2c coating and is, therefore, the only one shown here (Figure 4).

### 3.3. Analysis of Corrosion Solutions

**Figure 5:**
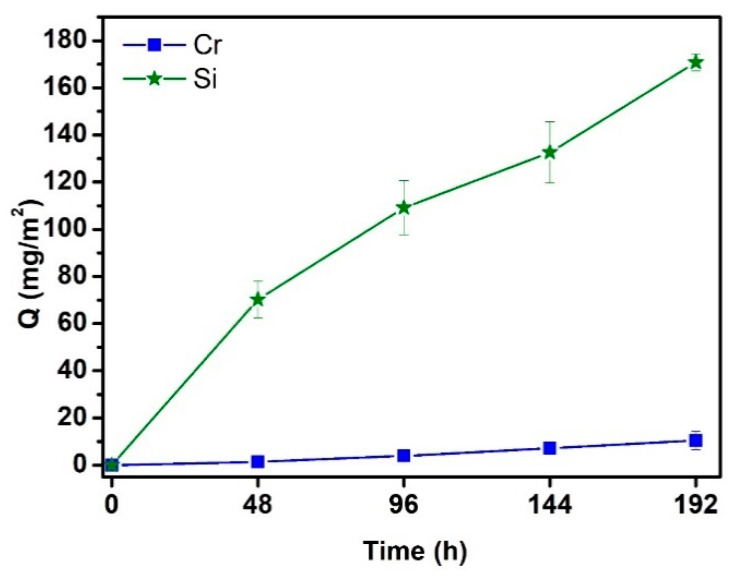
The amounts Q of elements (Si, Cr) leached from uncoated steel substrates in deionized water at 200 °C.

The amount of elements leached from the tested samples to the corrosion medium was determined by ICP-OES. Stainless steels contain Fe, C and at least 11 % of Cr, the element responsible for their corrosion resistance. In the case of uncoated steel (Figure 5), only the amounts of leached Cr and Si were considered because other elements contained in the steel were below the detection limit of the applied analytical method. Si was detected in the corrosion solutions, as Si, a common element in materials for elevated temperature applications, is also included in the composition of the studied ferritic AISI 441 stainless steel.

**Figure 6:**
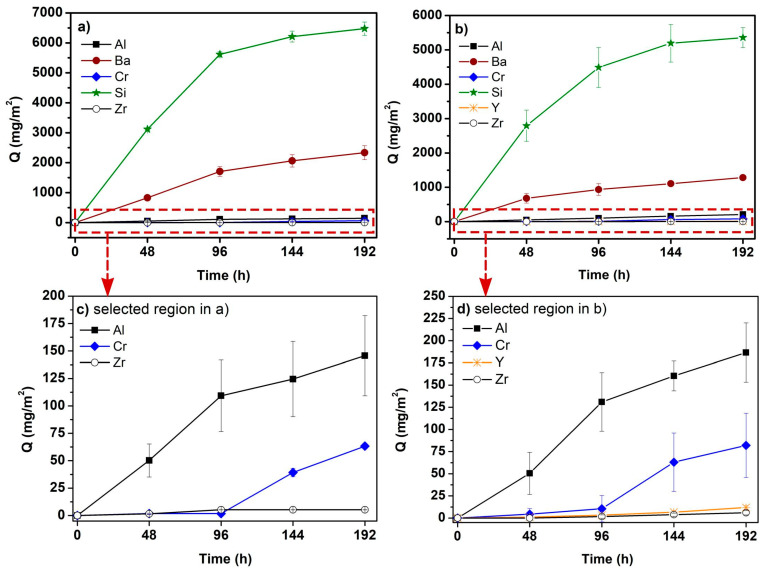
The amounts Q of elements (Si, Ba, Al, Zr, Y, Cr) leached from tested coatings in deionized water at 200 °C: (**a**) C2c coating; (**b**) D4 coating; (**c**) selected region in (**a**); (**d**) selected region in (**b**).

As for the coatings, analysis of the corrosion liquid by ICP-OES confirmed the presence of Ba, Al, Si, Zr, and Cr in the solution (Figure 6a–d). As with the uncoated steel substrates, the concentrations of Mn and Fe in deionized water were under the detection limit of ICP-OES. As can be seen in Figure 6c,d, only a negligible amount of Zr and Y in the corrosion solution was also observed. Very low concentrations of Zr detected in the corrosion solution suggest a high chemical durability of the YSZ used as a ceramic filler. Comparable amounts of Al were leached from both coatings to the solution at the applied quasi-dynamic conditions. Therefore, we suspect the AYZ filler was not the source of Al (it is contained only in the D4 coating) but the Al originates from barium aluminosilicate glass frits. These observations also suggest that AYZ filler is resistant to hydrothermal corrosion attack under the applied test conditions. As can be seen in Figure 6c,d, a small amount of Cr was identified in the corrosion solutions after 48 h and 96 h due to the outward diffusion of the Cr through the PDC coating. However, Cr content in both solutions dramatically increased after 144 h of corrosion test. This can be attributed to the faster outward diffusion of Cr ions probably due to higher occurrence of micropores and microcracks in the coatings that started to form after 144 h of exposure to the corrosive environment. Moreover, a higher content of Cr in the corrosion medium for both tested coatings was detected compared to the solution in which the uncoated steel was tested (Figure 5). The differences in Q values for Cr can be explained by the diffusion and release of Cr from steel and simultaneous precipitation of corrosion products containing Cr at the uncoated steel surface. In the case of the tested coatings, Cr remained dissolved in the corrosion medium since the coatings acted as a barrier for Cr migration back to the steel surface and prevented its precipitation.

From Figure 6 it is evident that the *Q* values for Si are significantly higher than the values for Ba and other leached elements. Moreover, the amount of Si and Ba leached into the corrosion media was found to be higher for the C2c coating than for the D4 coating. The total amount of Si identified in the solution is probably the sum of the contributions of Si leached from the glass frits, steel substrate, bond-coat as well as from the PDC matrix. In both coatings, the content of Si leached into the solution grew quickly in the first 96 h of the corrosion test, then the dissolution reaction slowed down as the state of saturation was attained and the precipitates were formed at the coating surface. We suppose that the amount of released Si increased even further, since existing and newly forming micro-cracks or micro-pores acted as weak points through which the corrosive medium can pass, thus causing further dissolution from these places. As a result, equilibrium was achieved and accompanied by a precipitation of reaction products at the coating surface.

The pH values of corrosion solutions were determined before and after corrosion tests and are summarized in Table 3.

The deionized water used in this study had a starting pH of 7.04 (19.5 °C) which increased upon the hydrothermal corrosion of the coated samples. In the time interval up to 96 h during the corrosion tests, the pH value of the corrosion solutions was found to be higher than 8 for both C2c and D4 coating tests. Considering the chemical composition of the two tested polysilazane-based (Si–C–N–O) coatings, this indicates that hydrolysis reactions in the PDC matrix occurred upon corrosion leading to the formation of silica and release of ammonia. Thus, we propose that the following process describes the hydrothermal corrosion of PDC-based coatings investigated in this work (2) (equation not balanced):

Si_x_C_y_N_z_O + H_2_O → xSiO_2_ + yCH_4_ + zNH_3_ (2)

In the first step, Si–N and Si–C bonds are attacked by deionized water accompanied by the formation of silica and a release of methane and ammonia, which is a weak base highly soluble in water [35]. This is in agreement with the increase of the pH values of the corrosion solutions during the corrosion tests. In a second step, water reacts with Si–O bonds, and silica dissolution occurs according to Equation (3):

SiO_2_ + H_2_O → Si(OH)_4_ (3)

Based on the results shown above, we assume that Si–N bonds in the investigated polysilazane-based coatings were attacked by hydrothermal corrosion while Si was released and present in the form of soluble Si(OH)_4_ in the corrosion solution. Considerably high concentrations of Si released into the corrosion medium from both tested coatings (Figure 6) further support this observation. However, as mentioned earlier, Si detected in the solutions could originate not only from the cleavage of Si–N and Si–C bonds, but also from the dissolution of the glass frits used as fillers in the coatings. However, the presence of a glassy phase in the coating structure is important for the corrosion protection due to the additional protective barrier provided by the glasses. The amounts of Al and Ba, along with Si, released to the solution likely shift the equilibrium towards precipitation within a short time interval. Because of this, the solution becomes saturated with respect to some secondary phases, which in turn precipitate at the coating surface in the form of insoluble barium aluminosilicate crystals, as confirmed by EDXS analysis of the coating surface after 192 h of corrosion tests (Figure 4). This could lead to a decreased rate of active corrosion for the studied coatings. A comparison of the corrosion behavior of our samples with other PDC glass/ceramic coatings is difficult to perform because of the lack of standard procedures in the corrosion testing of this type of protective coatings under hydrothermal conditions. However, similar behavior was observed in the case of SiC, Si_3_N_4_ or SiOC-based material [33,34,36]. For instance, SiOC-based ceramic nanocomposites were investigated with respect to their hydrothermal corrosion behavior at 250 °C. The results show an active corrosion behavior, i.e., silica was leached out of the samples [34].

### 3.4. Identification of Corrosion Products

**Figure 7:**
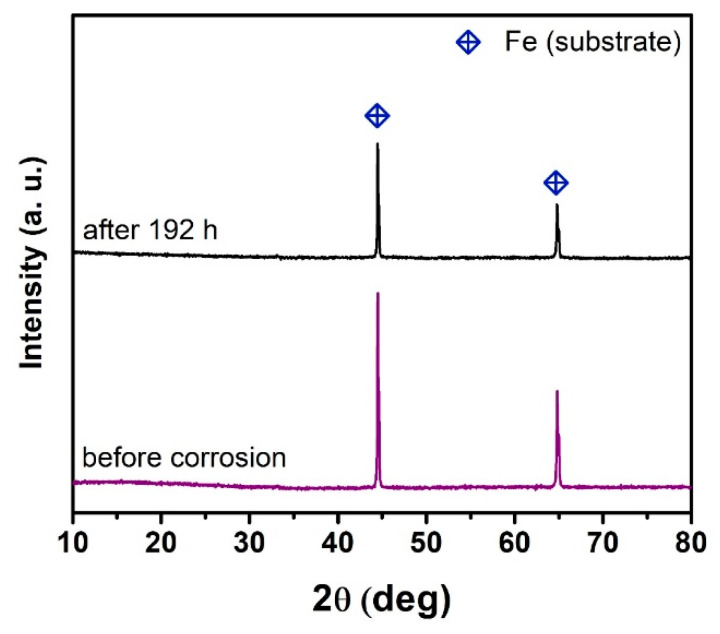
X-ray diffraction (XRD) patterns of uncoated steel before and after 192 h of corrosion tests.

XRD patterns of stainless steel before and after corrosion tests at 200 °C in deionized water are shown in Figure 7. In the steel substrates before and after corrosion tests, only the Fe phase (PDF-96-901-3474) was identified from the XRD patterns. No diffraction peaks belonging to the newly formed corrosion products were detected. However, the corrosion systematically led to a decreased amount of the Fe phase with increasing time of corrosion. This explains the gradual formation of corrosion products covering the steel surface, thereby reducing the intensity of the diffused signal from Fe.

Literature data [37,38] suggests that the oxide scale formed at the stainless steel surface in hot aqueous solutions exhibit a duplex structure with a Cr-enriched inner layer while the outer layer is Fe-enriched. Chromia Cr_2_O_3_ inner layer acts as a diffusion barrier for other elements (e.g., Fe, Ni), which prevents the metal from further corrosion [39]. For a Cr_2_O_3_ layer to be protective, it must be dense and continuous and cover the entire metal surface. However, the chemical compositions of the steel, its microstructure, and the service environments are the major factors affecting the formation of the passive oxide film. It is well known that corrosion of stainless steels is accelerated in atmospheres containing H_2_O.

**Figure 8:**
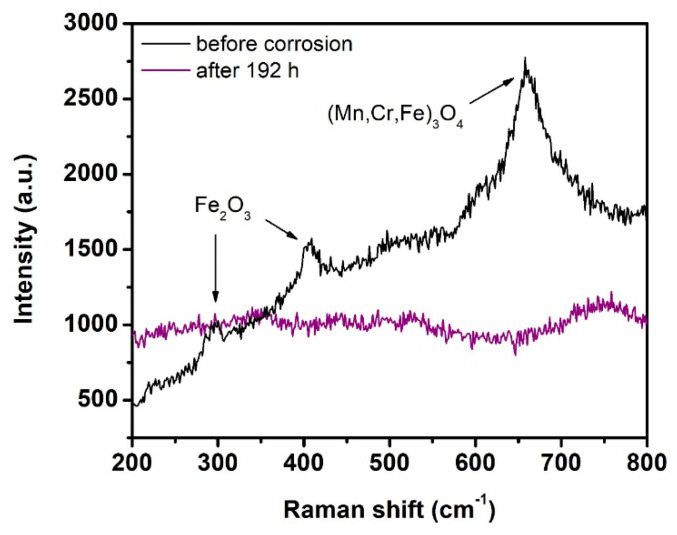
Raman spectra of uncoated steel substrates before and after 192 h of corrosion tests.

In order to identify the corrosion products formed at the steel surface after the corrosion tests, the specimens were also analyzed by Raman spectroscopy, as shown in Figure 8. The most intense feature in the spectrum of the uncoated steel after 192 h of corrosion tests was observed at ~660 cm^−1^ and corresponds to (Mn, Cr, Fe)_3_O_4_ spinel [40]. The bands around ~295 cm^−1^ and ~405 cm^−1^ were attributed to Fe_2_O_3_ [41,42]. The Raman bands of Cr_2_O_3_, typically appearing at ~550 cm^−1^ [40,43], were not found in the measured results. This indicates that crystalline Cr_2_O_3_ was absent or only present at low concentrations. Therefore, two hypotheses of the formation of corrosion products on the steel surface were considered. First, the protective Cr_2_O_3_ scale did not form in the initial corrosion stage, and it could not provide effective protection of the steel from future corrosion. The second hypothesis is that the protective Cr_2_O_3_ layer was formed in the initial time interval according to the reaction (4). However, at some point it lost its protective behavior due to the reaction with the deionized water resulting in the formation of chromium hydroxides. Both hypotheses correlate well with the absence of Cr_2_O_3_ peak in the XRD as well as in the Raman spectrum of corroded steel substrate. Furthermore, we assume that the deionized water reacts with the diffusing metallic species (Cr, Mn and Fe) according to the reaction (5) [44]:

2Cr + 3H_2_O → Cr_2_O_3_ + 3H_2_ (4)

3(Mn, Cr, Fe) + 4H_2_O → (Mn, Cr, Fe)_3_O_4_ + 4H_2_ (5)

Based on the Raman results, we can conclude that a majority of the corrosion products are a mixture of Fe_2_O_3_ and Mn, Fe, Cr spinels, as reported by other authors for Fe–Cr ferritic steels tested in water-containing atmospheres [43,44].

**Figure 9:**
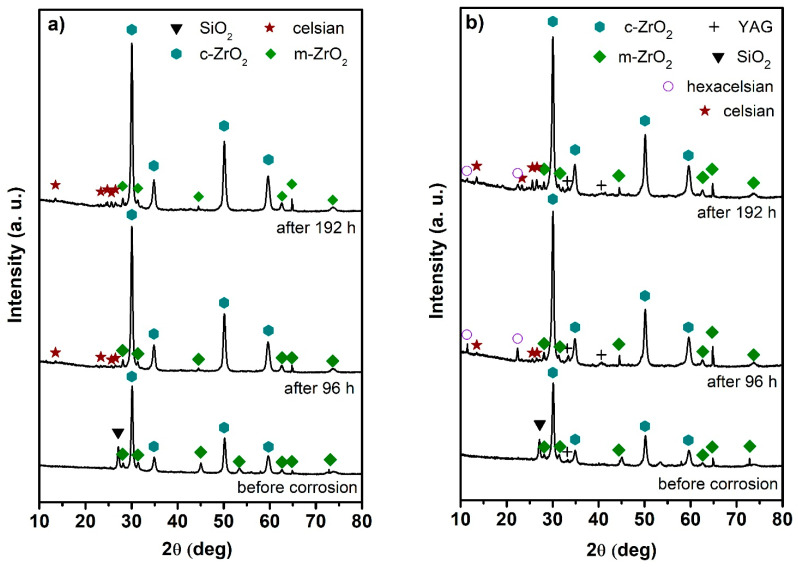
XRD patterns of the coatings before and after corrosion tests. (**a**) C2c coating; (**b**) D4 coating.

In the coated samples, XRD was used to detect any secondary phases that could result from chemical reactions between the components of the steel substrate, coating, and corrosive agent. In both tested coatings, the dominant phases detected after pyrolysis by XRD are monoclinic (PDF- 96-901-6715) and cubic ZrO_2_ (PDF-96-210-1235). Moreover, a peak located near the most intense diffraction peak of cubic ZrO_2_, assigned as SiO_2_ (quartz, PDF-96-901-2602), also appeared in the coatings, probably as a result of crystallization of the glass frit during pyrolysis. In the D4 coating, yttrium-aluminum garnet (YAG, PDF-96-431-2143) was also identified. This phase originates from the polycrystalline AYZ precursor powder used as passive filler. After 96 h of corrosion tests, new diffraction peaks are observed in both coatings, which are assigned to the crystalline phase BaAl_2_Si_2_O_8_ (celsian, PDF-96-201-3138). This is in accordance with the results of EDXS analysis of the precipitates formed at the coating surface. It can be noted that after 96 h of corrosion testing, a new phase, namely BaAl_2_Si_2_O_8_—hexacelsian (PDF-01-088-1049), a polymorph of celsian—is detected in the D4 coating. With increasing time, the XRD patterns show a decrease in the peak intensities of this phase (Figure 9b). The intensity of the diffraction peaks corresponding to cubic ZrO_2_ increased with the time of corrosion tests, while the SiO_2_ peak totally vanished after 48 h of exposure to the corrosion medium. This effect can be explained by the incorporation of SiO_2_ into other crystalline/amorphous phase and/or to the dissolution of the silica to the corrosion medium according to Equation (3).

Considering the chemical composition of the fillers used and the tested PDC coatings, and the corrosion products found on the corroded surfaces, it was deduced that the chemical reactions (6)–(10) likely occurred during corrosion test in deionized water at 200 °C:

BaO + Al_2_O_3_ → BaAl_2_O_4_ (6)

2BaO + SiO_2_ → Ba_2_SiO_4_ (7)

Ba_2_SiO_4_ + 3SiO_2_ → 2BaSi_2_O_5_ (8)

BaAl_2_O_4_ + 2SiO_2_ → BaAl_2_Si_2_O_8_ (9)

BaSi_2_O_5_ + Al_2_O_3_ → BaAl_2_Si_2_O_8_ (10)

Since the presence of these transient phases (BaO, BaAl_2_O_4,_ Ba_2_SiO_4_) was not detected in the XRD patterns, it was hypothesized that they reacted with the glassy phase or at other places in the coating locally enriched in SiO_2_ and Al_2_O_3_ as soon as they were formed to form the main corrosion product, BaAl_2_Si_2_O_8_. A large part of the coating surface was then covered by celsian precipitates, with an additional very small amount of residual hexacelsian crystals on the D4 coating. However, the formation of barium aluminosilicates by reactions of other components contained in the coatings or in the corrosion solution cannot be completely ruled out.

**Figure 10:**
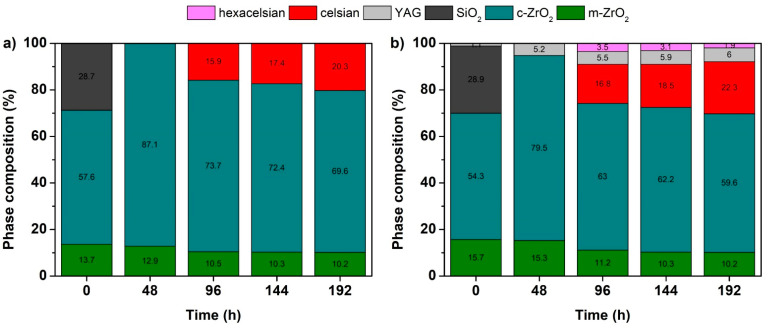
Time dependence of phase composition determined by the Rietveld refinement of XRD patterns acquired from: (**a**) C2c coating; (**b**) D4 coating.

Rietveld refinement of XRD data was used for the semi-quantitative analysis of the phase composition of the tested coatings. The time dependences of the phase composition of both C2c and D4 coatings are shown in Figure 10. The results indicate that the content of monoclinic ZrO_2_ slightly decreased with increasing time. As shown in the XRD patterns (Figure 9), the SiO_2_ phase disappeared in the early stage of corrosion testing. In contrast, an increasing content of celsian phase can be observed as a consequence of the chemical reactions between the individual components of the layers and/or elements leached to the corrosion medium.

### 3.5. Microstructures of Polymer-Derived Ceramic (PDC) Coatings

**Figure 11:**
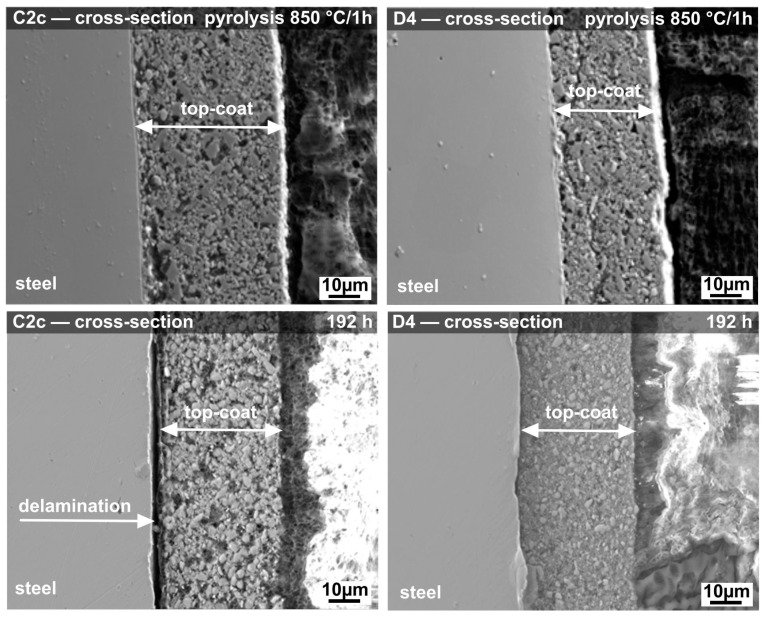
SEM cross-sections of C2c and D4 coatings before and after corrosion tests.

SEM/EDXS analysis was used for a detailed study of the cross-sections of coated samples before and after corrosion tests (Figure 11). Parchovianský et al. [30,45] have already described the microstructure of the PDC coatings studied in this work after pyrolysis. Results of these investigations are briefly described here. After pyrolysis in air at 850 °C for 1 h, homogeneous and almost dense protective coatings, with only small pore sizes, were prepared. Both coatings, C2c and D4, showed good adhesion: no gaps or cracks propagating along the metal/coating interface were detected. On steel exposed to ambient environment, a natural oxide layer with chemically bonded water is always present. Because of the high reactivity of Durazane 2250 with hydroxyl groups, steel forms direct metal–O–Si chemical bonds with the precursor-based bond-coat, leading to strong adhesion [17]. As indicated by SEM and EDXS elemental mapping [30], the coating microstructure is composed of three main constituents: evenly distributed original filler particles, and residual porosity aggregated in the amorphous PDC phase. The coating C2c is characterized by higher residual porosity. However, such a microstructure with residual porosity is beneficial for the thermal stability of the coatings, as it contributes to the reduction of residual stresses during heating and cooling cycles [46].

More promising results after corrosion tests were observed for the D4 coating, which contains the polycrystalline AYZ powder as passive filler. The D4 coating showed lower porosity than the C2c coating (Figure 11). SEM cross-sectional images also showed good coating adhesion, even after l92 h of corrosion tests. Overall, no significant visible corrosion damage was observed on D4 coating after the tests, confirming it acted as an efficient protection system. In the case of the C2c coating after corrosion tests, a significant increase in the porosity of the layer accompanied by the growth of pores was observed. Moreover, spalling of the C2c coating occurred after 192 h of exposure to deionized water.

There are several factors that could cause the better corrosion performance of the D4 coating. The first is the composition, the lower content of YSZ filler and the addition of polycrystalline AYZ powder in the D4 coating structure. The lower weight loss during the corrosion tests of the D4 coating could also be influenced by the use of AYZ filler, which obviously acts as reinforcing phase, resistant to the attack of deionized water and improving the hydrothermal stability of the PDC matrix. The better corrosion resistance of the D4 coating can be also attributed to the different microstructure of the coatings. The addition of the AYZ powder with irregular and angular particles helped create a solid and rigid structure which allowed outgassing of the preceramic polymer pyrolysis products from the system, thereby effectively reducing the size and amount of pores after pyrolysis. The absence of larger pores, and thus an increased density, led to a significantly more compact coating in comparison to the C2c composition pyrolyzed under the same conditions. The defects of a critical size in the C2c coating could induce macroscopic failure and thus reduce the adhesion strength resulting in the delamination of the ceramic layer during corrosion testing. The increased occurrence of pores and cracks in the C2c coating also allowed a faster penetration of deionized water through the coating to the metal substrate causing a subsequent spallation and disintegration of the layer. The contents of preceramic polymer (source of Si) and glass frits (source of Si and Ba) in both coatings are identical and cannot explain the difference of the leached amounts of these elements to the corrosion solutions. Therefore, the higher number of pores is likely to be responsible for higher dissolution rates of Si and Ba in deionized water, which could cause faster recession and failure of the C2c coating during the corrosion tests. Based on the observations shown above, it is proposed that the highly porous microstructure of the C2c coating is the main reason of failure for this coating.

**Figure 12:**
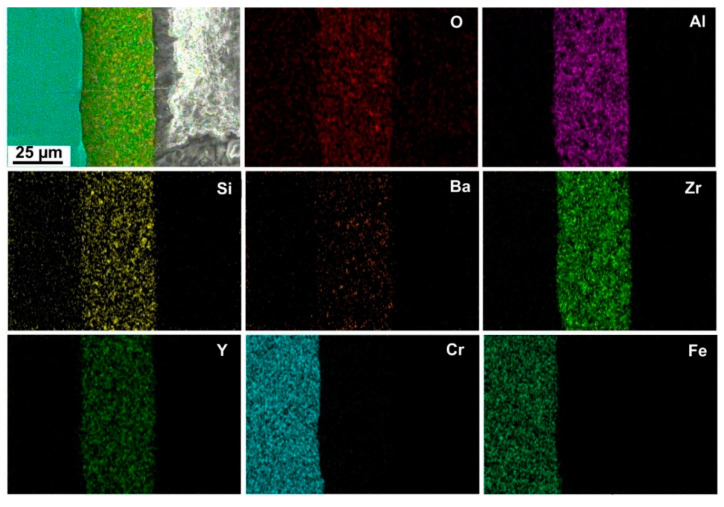
SEM/EDXS cross-sections of the D4 coating after 192 h of corrosion tests.

EDXS mapping was performed on the cross-section of the D4 coating (Figure 12). The EDXS maps identified a homogeneous distribution of Zr, Y, Si, Ba, Al, and O in the top-coat. The presence of Fe and Cr is clearly seen in the stainless steel substrate. As confirmed by ICP-OES analysis of the corrosion solutions, no diffusion of Fe from the substrate through the coating was observed. This is also shown in the EDXS map where the presence of Fe ends exactly at the steel/coating interface. However, EDXS cross-sectional analysis revealed the presence of a small amount of Cr in the top-coat, which likely diffused out of the steel during the tests. This is also consistent with the presence of Cr in the corrosion solution detected by ICP-OES. No corrosion products were observed at the stainless steel/top-coat interfaces after corrosion tests.

## 4. Conclusions

In this work, the hydrothermal corrosion resistance of ferritic stainless steel and two PDC-based coatings was studied systematically in deionized water. Two types of corrosion product morphologies were found on the exposed uncoated steel surface. Most of the surface was covered by a thin layer of rod-shaped crystallites identified by Raman spectroscopy as a mix of Fe_2_O_3_ and (Mn, Cr, Fe)_3_O_4_ spinels. Occasionally, Fe- and Cr-enriched globular crystallites were found on the corroded steel surface after the corrosion tests. In the case of the coated samples, the results of weight gain measurements together with SEM and ICP-OES indicate that a state of saturation was achieved in the early stage of the dissolution reactions. These reactions were followed by the precipitation of spherical-shaped BaAl_2_Si_2_O_8_ crystals. M–ZrO_2_, c–ZrO_2_, YAG, and BaAl_2_Si_2_O_8_ –hexacelsian, celsian phases were identified from XRD patterns of the corroded PDC coatings. The D4 coating exhibited better protective properties than the C2c coating in deionized water under quasi-dynamic conditions at 200 °C. No significant corrosion damage was observed for the D4 coating, but the C2c coating showed a highly porous structure, delamination of the protective layer and the loss of corrosion resistance. The results of this study indicate the high potential of the investigated PDC coating with AYZ passive filler for applications in harsh environments, such as hydrothermal corrosion conditions.

## Figures and Tables

**Table 1:** Compositions of prepared composite coatings (vol. %) and their coefficients of thermal expansion (CTE).

Compositions	Durazane1800	YSZ	Glass G018-281	AYZ	CTE (10^−6^/K)
C2c	30	35	35	-	10.1
D4	30	17.5	35	17.5	9.4

**Table 2:** Basic properties of filler materials.

Passive Fillers	d50 (µm)	ρ (g/cm^3^)	CTE (10^−6^/K)
8YSZ	0.5	6.1	11.5
AYZ	1–10	4.6	8.6
Glass G018-281	0.5–5	2.7	12.1

**Table 3:** Evolution of the pH values of the corrosion medium with increasing time of corrosion.

Sample	48 h	96 h	144 h	192 h
Steel	7.05	7.12	7.10	7.11
C2c	8.90	8.30	7.55	7.46
D4	8.64	8.24	7.51	7.43

## Data Availability

Not applicable.
